# The Influence of Taste Liking on the Consumption of Nutrient Rich and Nutrient Poor Foods

**DOI:** 10.3389/fnut.2019.00174

**Published:** 2019-11-15

**Authors:** Djin Gie Liem, Catherine Georgina Russell

**Affiliations:** ^1^Deakin University, Geelong VIC, Australia; ^2^Centre for Advanced Sensory Science, School of Exercise and Nutrition Sciences, Deakin University, Geelong VIC, Australia

**Keywords:** taste, consumer, sensory, food, nutrition

## Abstract

Repeated consumption of high-energy nutrient poor foods can lead to undesirable health outcomes such as obesity. Taste plays an important role in food choice, and a better understanding of the links between the taste of foods, individual taste preferences, food choices, and intakes will aid in our understanding of why some people might select and consume unhealthy foods. The present review focuses on three main questions: (1) do nutrient poor and nutrient rich foods significantly differ in taste profile? (2) are humans predisposed toward developing a liking or preference for certain taste profiles? (3) how are individual variations in liking of the basic taste qualities related to long term food intake and adverse health outcomes such as obesity? Results indicated that nutrient poor foods were likely to be sweet, salty and fatty mouthfeel, while the taste profiles of nutrient rich foods were diverse. Although humans are born with a universal liking for sweet and aversion for bitter taste, large individual differences exist in liking of all the basic taste qualities. These individual differences partly explain differences in short term intakes of foods varying in taste profiles. However they fail to sufficiently explain long term food choices and negative health outcomes such as obesity. Future studies should focus on how the full sensory profile of food which includes taste, smell and texture interacts with individual characteristics (e.g., taste or health motivations, taste preferences) to affect consumption of nutrient rich and nutrient poor foods.

## Introduction

Overweight and obesity continue to be a major public health challenge in economically developed nations. Approximately 20% of adults in OECD countries are obese which varies from 3.7% in Japan to as high as 27.5% in Australia (1) and 39.8% in the USA (2, 3). In general, obesity is positively correlated with the development of chronic diseases such as diabetes and heart diseases (4, 5), as well as social psychological challenges (6). One of the major contributors to the overweight/obesity epidemic is the food environment and associated dietary choice (7). Dietary patterns that are associated with higher levels of overweight/obesity are often (but not always) characterized by high levels of energy (calories) intake, coupled with low levels of nutrients (7). That is, the foods consumed as part of these dietary patterns are often low in nutrient density.

Nutrient rich foods can be described as “foods which provide substantial amounts of nutrients for relatively few calories, or foods which provide fewer calories than nutrients” (8). Nutrients in this context refer to health promoting nutrients such as protein, fiber, vitamins A, C and E, calcium, magnesium, iron and potassium, and does not refer to non-health promoting nutrients like saturated fat, sodium, and added sugar. Nutrient density can be reflected by the Nutrient Rich Food (NRF) index which is calculated as the sum of percentage daily values for 9 nutrients that should be consumed in higher quantities (e.g., protein; fiber; vitamins A, C, and E; calcium; magnesium; iron; and potassium) minus the sum of 3 nutrients which should be limited (e.g., added sugar, saturated fat, sodium), with all daily values calculated per 100 kcal and capped at 100% (9). Diets with foods with a high NRF scores (i.e., nutrient rich foods) are positively linked to a higher consumption of foods and nutrients which are encouraged for better health, and a lower energy intake overall (10). In contrast, the over consumption of foods with a low NRF score (i.e., nutrient poor foods) has been associated with weight gain and subsequent negative health outcomes such as diabetes (8, 11). This is not surprising given that, in general, nutrient density (as in its definition) is negatively correlated with energy density (9). In order to increase the consumption of nutrient rich food and to decrease the consumption of nutrient poor foods, it is important to understand what affects the consumption of such foods (12).

A wide range of studies shows that food *liking* is one of the most important driver of food consumption (13–21). Food liking, includes liking of the basic taste qualities [e.g., sweet, sour, bitter, salty, umami and fat (22, 23)]. In this paper we focus on the role of taste liking in affecting choice and consumption of both nutrient rich foods and nutrient poor foods. In order to understand the high levels of consumption of nutrient rich foods and lower levels of consumption of nutrient poor foods, we will address the following questions (1) do nutrient poor and nutrient rich foods significantly differ in taste profile? (2) are humans predisposed toward developing a liking or preference for certain taste profiles? (3) how is individual variation in liking of the basic taste qualities related to long term food intake and adverse health outcomes such as obesity?

To answer these questions we will first discuss the taste profiles of nutrient poor and nutrient rich foods and diets. Next, we will discuss how taste liking develops in humans and how this is related to the consumption of nutrient rich and nutrient poor foods. Lastly, we will propose a series of recommendations, which aim to increase the consumption of nutrient rich foods and decrease the consumption of nutrient poor foods.

## Taste Profiles of Nutrient Rich and Nutrient Poor Foods in the Current Foods Supply

Today's modern industrialized food supply is dominated by energy rich, nutrient poor foods (24). These foods are composed of high levels of sugar, saturated fat and sodium and are highly processed and easily accessible due to high volumes, low prices and ease of consumption (e.g., no elaborated preparation required). Data from Australia suggest that the availability of oils for cooking and food production increased by more than 600% from 1961 to 2009 (25). In addition, data from another Australian study showed a large increase in volume and value (around 580%) of imported sweetened products between 1988 and 2010, while exports of similar goods were minimal in comparison (26). Such increase in accessibility of high energy nutrient poor ingredients and foods is associated with the increased consumption of such foods (24, 27). Studies from Western countries suggest that the majority of energy average people in economically developed nations consume now comes from these high (added) sugar, sodium and saturated fat rich foods (12, 28–30).

Sugar (31), fat (32), and sodium (33, 34) significantly impact the taste profile of food (35). Mapping this against the NRF index, it can therefore be hypothesized that individual nutrient poor and nutrient rich foods have different taste profiles. Likewise, it can be hypothesized that healthy diets, which are dominated by nutrient rich foods, have different taste profiles compared to unhealthy diets, which are dominated by nutrient poor foods. These assumptions are explored in the following paragraphs.

Although it makes intuitive sense, there are at least four challenges with the assumed taste-nutrient relationships posited above. Firstly, not all nutrients can easily be sensed. For example, sodium in bread is less accessible to sodium sensing channels on the human tongue, than sodium on the surface of chips (33, 34). Therefore, bread can appear less salty than chips at the same sodium content. Secondly, modern advances have been able to decouple some sensory profiles and nutrient composition. For example, non-nutritive sweeteners provide sweet taste without the calories (36). Therefore, the nutrient content of foods may not match their perceived taste intensity.

A third challenge is that specific taste qualities are dominant in both healthy and unhealthy foods. For example, sweet taste occurs in both nutrient rich (e.g., fruits) and nutrient poor (e.g., sugar sweetened beverages) items. Similarly, bitter taste occurs in both nutrient poor (e.g., alcoholic beverages such as beer and wine) (37) and nutrient rich foods (e.g., cruciferous vegetables such as broccoli and Brussels sprouts (16, 38). The fourth challenge is that there are many taste-taste interactions which potentially disturb the taste-nutrient relationship (39). For example bitter taste suppresses sweet taste (40).

The relationship between the presence of particular nutrients such as sodium or sugar in foods, and their perceived taste intensity is therefore complex. In order to investigate the hypothesis that nutrient poor and nutrient rich food have different taste profiles, firstly foods need to be assessed on their macronutrient composition. Secondly, the same foods need to be sensory profiled by a group of trained human panelists. To this end, several scientists have established taste databases (35, 41–44). These databases contain foods and dishes which are commonly consumed in the country of interest and are assessed on their macro-nutrient profile. By systematically assessing these foods on the presence and intensity of basic taste qualities (e.g., sweet, sour, salty, bitter, and umami) and certain texture properties such as fatty mouthfeel, a food taste database can be established. It is important to note that these databases are mainly focussed on taste, rather than flavor, which is the combination of taste, smell and chemical irritation (45).

To our knowledge the first scientifically peer reviewed published attempt to establish such taste database, came from the Netherlands (43). In this study a small number of frequently consumed foods (*n* = 50) was sensory profiled by 19 minimally trained young consumers. The foods were assessed on the perceived intensity of sweet, sour, bitter, salty, and umami-taste, by following the Spectrum Method. In this method, panel members are first calibrated on using the same terminology (attributes) for the sensation of interest, by using reference standards and extensive discussions amongst the panel members, facilitated by the panel leader. Next, panel members are calibrated on the intensity of the generated attributes by using reference samples which provide a wide range of intensities of the attribute of interest (46). This methodology to establish a taste database has been repeated with more heavily trained adults (35), in-home panels (41), a higher variety of foods [e.g., 377 food items (35), 237 food items (44), 590 food items (41)], and additional attributes including texture attributes (e.g., hardness, moistness, cohesiveness of mass and fatty mouth feel) (35, 41). These databases provide the means to examine whether nutrient poor and nutrient rich foods can be characterized by particular taste profiles.

## Nutrient Rich Foods and Taste Profile

Despite the potential disparity between taste and nutrient content, as mentioned previously, across a number of studies in different countries it has been shown that taste significantly relates to specific macronutrient content in foods. That is, the sweetness of food is positively correlated with the presence of mono- and disaccharides (35, 42, 43, 47, 48), salty taste is positively correlated with the presence of sodium (35, 42, 43, 47, 48) and to some extent protein (35, 43). Umami taste is positively correlated with the presence of protein (42, 47) and sodium (35). Fat sensation is related to fat content (35, 42, 47). Sour taste is related to the presence of organic acids (47). It is important to note that the association between nutrient content and taste varies amongst foods. Where the association tends to be weaker in more complex foods (35).

Given the high presence of added sugar, sodium and fat in nutrient poor foods and the lower levels of these nutrients in nutrient rich foods, it is to be expected that nutrient rich foods have a lower taste intensity than nutrient poor foods. This is partly confirmed by studies from the Netherlands (44), and France (41). These studies suggest that nutrient rich foods have a rather diverse taste profile. Van Langeveld et al. (44) concluded that staple foods (e.g., bread, potato) and what are generally considered to be nutrient rich foods, such as vegetables and fish, were overrepresented in the neutral taste cluster (e.g., these foods were perceived as having a low taste intensity of all basic taste qualities). However, other nutrient rich foods such as nuts, fruits, meat, and milk were mostly classed as salt/umami/fat, sweet/sour, sweet/fat, respectively. This is in line with data from France whereby fruits were mostly present in the sweet/sour and to some extent bitter clusters. Interestingly, in this French study, vegetables were mostly represented in the salt, umami, sour and bitter clusters which most likely represents the way many consumers in France prepare their vegetables (41).

The taste profile of nutrient poor foods is more consistent than that of nutrient rich foods. In the Netherlands (48) as well as in France (41) is was found that nutrient poor foods mainly have a taste profile which can be summarized as sweet, salty and fatty mouthfeel). Because of the high impact of salt/umami, sweet taste and fat taste on the sensory profile (35) and given the homogeneous taste profile of nutrient poor foods, it can be hypothesized that diets which are high in taste intensity are more likely to be nutrient poor. This indeed seem to be the conclusion from a Dutch study which assessed the taste patterns of different diets, including a diet based on the Dutch dietary guidelines (49). It was concluded that the energy derived from a diet based on the Dutch dietary guidelines mostly comes from neutral/bland tasting foods (49).

## Innate Preference for Taste Profile of Nutrient Poor Foods

Now that we have more insights in the taste profile of nutrient poor foods, a related question becomes “does the taste profile of nutrient rich and nutrient poor foods affect their consumption”? In this section we specifically focus on sweet, salty and fatty taste as those tastes that are characteristic of nutrient poor foods. In addition, we focus on bitter and sour taste, because nutrient rich foods such as cruciferous vegetables have a bitter note (16) and many fruits have a sour note to them (50). However, it needs to be mentioned that other sensory aspects of food such as smell, which plays an important role in increasing our appetite [see (14) for review], and texture, which plays a role in the amount we consume [see (51) for review], also play a major role in food intake but are not explicitly considered here.

There are several biological underpinnings to the sense of taste. At birth humans are already equipped with a taste apparatus which can distinguish between sweet, sour, and bitter taste as evidenced by distinct facial expressions of newborns when exposed to sweet, sour and bitter tasting substances dissolved in water (52–54). Not only can infants detect these tastants, as judged by facial expression, the sucking responses and to some extent intake of these tastants follow the positive (e.g., sweet taste) or negative (e.g., bitter and to some extent sour taste) facial expressions (52–54).

Most research in the area of taste and infants (and children) has been focussed on sweet taste (55–57). Newborns appear to have a clear preference for sucrose solutions over plain water, as evidenced by relaxed facial expression, increased sucking responses and intake (54). Supposedly liking for sweet taste provides an evolutionary advantage for humans as sweet taste is (in nature) related to energy content, which is needed for growth and development (31). The liking of sweet taste also facilitates the ingestion of breastmilk, which has a sweet taste profile. It has been consistently found that children from a range of countries prefer higher intensities of sweet taste than adults (55) highlighting a clear innate bias toward sweet taste in infants and children.

The perception of salty taste is thought to go through different stages during development [see (58) for review]. Shortly after birth 1 to 4 days old infants seem to be mostly indifferent to salty taste which is likely due to the postnatal maturation of specific central and/or peripheral mechanisms underlying salt taste perception (59). At ~4–6 months after birth, infants appear to have a preferential response to salty water over plain water (54, 60) without the need for prior exposure to salty taste. This suggests an innate preferential response to salty taste. Such preference for salty taste is reflected in infants' increased consumption of salty baby cereal over plain baby cereal (61). When infants grow into early childhood (about 3 years of age), they show adult like rejections to salted water, but the liking for salt in culturally accepted salty foods remain high (60). This suggests that liking for salt is food specific and partly learned by exposure.

Whether humans have an innate preference for fat taste remains unclear. Several studies investigated infants' sucking and/or the ingestive response of infants to breast milk (62) or infant formula (63) with varying fat content. These early studies argue against the presence of an innate liking for fat. A more recent study investigated the development of fat liking by following 3 months infants up to the age of 20 months and also concluded that similar to newborns, 3 months old infants were mostly indifferent to the taste of fat emulsions (64). It seems from available evidence, then, that the liking for fat is mostly learned through dietary exposure, a process which may also be affected by the higher energy density of higher fat foods.

Bitter taste is clearly rejected by infants as evidenced by negative facial expressions (e.g., mouth gaping and wrinkling of regions on the forehead). There is little doubt that newborns are able to detect bitter taste (52–54), however they do not seem to diminish intake in response to the sensation of a bitter substance. That is, sucking responses and ingestion in response to a bitter solution are not different from those to water (65, 66). An alternative explanation is that infants dislike plain water. In nature, many toxic substances taste bitter, so it seems to fit a natural survival instinct to reject any foods which are bitter (67).

It is generally thought that strong sour solutions are disliked by newborns, although the facial response to sour solutions (e.g., lip pursing) is remarkably different from the facial response to bitter solutions (e.g., mouth gaping, wrinkling of the regions of the forehead) (52–54, 66). The combination of lip pursing and sucking, seen typically in response to sour tasting substances, may result in compressing the cheeks against the gums, which stimulates salivary flow in the oral cavity. In adults it has been suggested that the increased flow and buffering capacity of saliva neutralizes sour tasting substances (68, 69). Infants' ingestive responses to sour taste do not provide a clear picture indicating a clear rejection. Although a mixture of sucrose and citric acid resulted in lower intake than a sucrose solution in some studies, it does not take into account the suppression of sweet taste by citric acid. Moreover, no difference in the infants' ingestion has been observed in response to water and water with added citric acid (65). In infants close to one and a half years of age it has been shown that about a fifth to one third of these infants have a preference for high concentrations of citric acid in a sugar solution. These infants were more likely to consume fruit than those infants who did not express this preference for high sour tasting sweet solutions (70). Similar observations have been made in children (71–73). This suggests that sour taste preferences directly influence food consumption.

When infants grow into early childhood, the preference for sweet taste (74–76) and the aversion for bitter taste remains high (77, 78). Research conducted in a range of countries suggests that liking for sweet taste is significantly higher in children than adults (55). Liking for sweet seems to coincide with rapid changes of grow during childhood (79), which supports the hypothesis that children's high liking for sweet taste is functional for the rapid stages of growth and development infants and children go through. In addition it has been suggested that children are more sensitive for bitter taste than adults, which might explain why children often reject the slightest note of bitter taste (57, 80) and children's consumption of bitter tasting vegetables can be a struggle (16). When children grow older, new flavors and tastes are added to their repertoire of acceptable foods, mainly through different learning mechanisms, as discussed below.

## Learned Taste Preferences

As noted above, humans have innate biological biases that, in the absence of other factors, may predispose them toward liking foods that are sweeter, fattier, and/or saltier and disliking foods that are bitter and possibly sour. However, taste liking arises due to the interacting effects of genetic predispositions and environmental factors. That is, although there are a number of innate taste biases that appear to be common to all humans, they cannot solely explain the wide variation in food/taste liking observed in populations (81, 82). Rather, individual differences in food/taste liking that are observed between individuals reflect interactions between innate biological characteristics and learning processes that occur over time.

Innate biological characteristics include those that are common to all humans such as a liking for sweet taste, as discussed above, along with variations between individuals in these characteristics [e.g., capacity to detect bitter taste (83, 84)]. Individual differences in biological characteristics may influence how individuals respond to the influences within their food environments, and therefore the tastes and foods that are liked or disliked. Evidence in children has shown the important influence of individual genetic differences on liking (85) as well intake of vegetables (86), although this effect seems to diminish in adulthood and older age (80, 87). Furthermore, twin research in adolescents suggest that unique family environmental influences succeed shared environmental influences in young adulthood (unlike in early childhood), highlighting that individuals within similar food environments respond in different ways (88), resulting in taste, and subsequently food, likes and dislikes that are unique to individuals (85, 89)

The ways in which non-shared environmental effects may manifest in unique taste and food liking is through the effects of the social and environmental context of consumption. These effects are also rooted in biologically based predispositions that result in rejection of novel foods (food neophobia), but also greater acceptance of foods with exposure and positive associations: Through the repeated pairing of foods with positive or negative stimuli individuals learn to like and dislike particular tastes and foods. This is why exposure and familiarity are key mechanisms explaining differences between individuals and populations in taste liking and disliking. Exposure can begin early, with flavors experienced *in utero* and in breast milk influencing liking of flavors/tastes in both shorter and medium terms (90–92). Exposure that is linked to positive affective tones can accelerate the effects of exposure on food liking of particular foods and sensory characteristics (93). In contrast, foods that are presented in ways that elicit negative affect can result in decreased liking of those foods and associated sensory characteristics. Parental use of nutrient poor foods, such as dessert foods, as food rewards, for instance, accelerates children's liking of those foods (94). In contrast, rewarding children for eating nutrient rich foods (e.g., vegetables), or pressuring children to eat them, decreases liking of such foods (95). What determines the nature of individual exposure to a range of tastes and foods, such as use of pressure and reward during parental feeding, is related to a wide range of biological, psychosocial and cultural factors (96, 97).

Also important are learned associations between sensory cues of foods and post-ingestive consequences (98). This works in two important ways: through the formation of food aversions associated with nausea or vomiting (99), and through positive associations between the sensory properties including tastes and flavors of foods that are more filling and satisfying (100). This is one mechanism by which the tastes of more energy dense foods can become liked, and bitter and sour tastes can become liked. However, although this mechanism has, in some instances, been demonstrated in relatively low energy density foods including fruits and vegetables (101) where additional energy is added to increase the overall energy density of the foods, it is more likely to be a mechanism helping to explain liking of foods that are naturally high in energy density, which is a characteristics of many nutrient poor foods.

As noted above, individual differences in biological characteristics can help to explain unique taste and food likes and dislikes. Important individual differences in biological characteristics include variations in taste receptors, which affects sensitivity to the various tastes and taste intensities; notably bitter taste. Greater bitter taste sensitivity has been linked to lower liking of cruciferous vegetables dine, along with greater sensitivity to sweet liking of foods, and lower liking of fatty foods that are strong tasting and sweet tasting (102, 103). However, other biological factors are also associated with taste and food liking, and these also appear to be linked to, and interact with, biological differences in taste acuity. For instance, cognitive approaches to eating such as food neophobia are associated with greater bitter taste sensitivity, as well as reduced exposure to, and liking of vegetables (104). Other differences in individual psychobiological characteristics such as temperament and personality, restraint and disinhibition, and reward circuitry also affect how individuals approach food and eating and, through learning mechanisms, taste and food liking (105).

Finally, it should also be noted that taste liking is not stable within individuals, and can vary with a number of factors including psychophysiological states, across the course of a meal, with hunger levels, mood/emotional state, and eating context and this can affect whether nutrient rich or poor foods are selected and consumed (106).

The interacting influences of unique and common biological factors with the unique characteristics of individual food environments produces a wide range of taste and food likes and dislikes. There are innate predispositions common to all humans that facilitate learned liking and consumption of nutrient poor foods (e.g., flavor-nutrient learning), and retard liking and consumption of some nutrient rich foods (e.g., food neophobia). Further, some individuals, for instance those who have higher sensitivity to bitter taste, are probably more susceptible to developing taste and food likes and dislikes that are consistent with consumption of nutrient poor foods.

## Relationships Between Taste Perception And Food Choice/Liking/Flavor Liking (in Different Individuals/Consumers)

So far we have discussed how nutrient poor foods have specific taste profiles, how the human biology is designed toward a liking of these typical taste profiles and how humans can learn to like foods associated with these taste profiles. But does individual variation in liking of these taste profiles alone lead to a higher consumption of foods with taste profiles commonly seen in nutrient poor foods? A further question is whether liking for particular taste profiles is related to adverse health outcomes.

## Sweet Taste and Dietary Intake

Several researchers have sought to find an association between sweet taste sensitivity, perceived intensity, sweet taste liking and intake of sweet tasting foods. A recent systematic review of 17 studies concluded that most studies which were reviewed failed to find an association between sweet taste sensitivity and dietary intake patterns (107). Also the potential relationship between perceived sweet taste intensity and intake is rarely shown (107). If anything there might be a negative association between perceived sweet taste intensity and energy and carbohydrate intake (108, 109), but again this has not consistently been shown.

The strongest and potentially the only association between sweet taste and intake is that of hedonics and intake. In particular, those studies that divided participants in either sweet likers group or sweet dislikers, used a more precise dietary intake tool (e.g., 24 h recall, 4-day weighed food record, 7 day diet record), and had sufficient sample sizes found statistically significant positive relationships between liking for sweet taste and dietary intake (107). That is, those who show a general liking for sweet taste consumed more energy from refined and total sugars (107), which are commonly nutrient poor foods. But as suggested earlier, sweet taste as such does not seem to correlate well with the energy in foods (44). If this holds true one would expect the association between sweet taste liking and obesity to be weak or non-existing. This indeed seems to be the case in that the majority of studies investigating the link between sweet taste perception/hedonics and obesity failed to find such a relationship (109, 110).

## Salt Taste and Dietary Intake

The relationship between salt taste liking and intake appears to be malleable. Longitudinal experimental studies in adults suggest that changing the salt content of foods is followed by a change in liking for salt taste as well as perceived intensity of salt taste. That is, a prolonged exposure 5 months (111), 12 months (112) to a low sodium diet resulted in a lower perceived salt intensity and liking for lower salt levels, compared to before subjects went on the low salt diet. Vice versa, when sodium intake increases, preferred levels of sodium in foods increase accordingly (113) [see (114) for review]. These studies suggest that salt taste perception and liking can be modified by changing dietary sodium intake. However, it needs to be noted that dietary sodium reduction in intervention trials are rather severe (24% dietary sodium reduction (111), 21% (112), whether smaller changes in sodium consumption would also result in a change in salt sensitivity and/or liking remains unclear.

When investigating natural variation in salt taste preferences, some studies found a positive association between preference for salty taste and consumption of sodium (115–117), whereas others did not see such correlation (118, 119). Such inconsistent results might be a partly caused by difficulties around the assessment of dietary sodium consumption and the likelihood that sodium intake is influence by more than just taste preferences [see Mattes (120)].

## Fat Taste and Dietary Intake

Recently fat taste has become of specific interest because of advances in the understanding of the perceptual mechanisms as well the association between fat taste sensitivity and consumption of fat (22, 23, 121, 122). It is important to note that fat taste refers to cellular responses to free fatty acids, rather than the cellular response to the most common form of fat in the diet (123). Observational studies as well as experimental studies have shown that fat taste sensitivity is negatively associated with the (short term) intake of dietary fat (122, 123), potentially this is caused by the influence of fat taste receptors on feelings of satiety. That is, when fat taste receptors (which are present throughout the GI track) are stimulated they trigger the release of various satiety hormones like GLP-1 and CCK (123) which generates a feeling of fullness. Some, but not all, studies found significant associations between fat taste sensitivity and obesity. That is obese people are more likely to be less sensitive to fat taste than lean counter parts (123, 124). The taste of dietary fat is also determined by texture properties. When taking the sensory perception of fat as a whole (e.g., taste and texture properties) some, but not all studies suggest a positive link between liking of dietary fat, fat consumption and obesity (122, 125–129).

Overall it can be concluded that although the taste profile of nutrient poor foods is rather consistent as judged by the basic taste qualities, there is a lack of consistent evidence which suggests that a taste liking of the basic taste qualities is what is driving long term food consumption leading to adverse health outcomes such as obesity. This can be partly due to the variety of methods used to measure liking for the basic taste qualities and they procedures which are followed to estimate dietary intake. In addition it is good to realize that food liking is not only determined by taste, but also by other food related properties such as smell, texture and appearance. Many studies have shown that liking of food as a whole, plays a major role in food consumption (13–21).

## Discussion and Recommendations

The taste profile of energy poor foods are naturally attractive for consumers. Although food liking as a whole (including taste, smell, and appearance) is an important driver of food choice, it is difficult to relate long term food choice of adults to the liking of specific basic taste qualities. This might partly be caused by methodological challenges in which basic taste qualities are often measured in isolation rather than its natural occurrence in foods. In addition taste preferences might play a different role during the life span. That is, children's food choices seem to be stronger related to basic taste preferences, than adults' food choices.

Humans seem to naturally like nutrient poor foods, and this can be reinforced by a range of environmental factors. At the same time segments of consumer can follow a healthy diet. This is not to say that these consumers do not care about how the food tastes, but other factors might make them more resilient to the temptation of taste or prevents consumers to choose the food they like the most. With regards to nutrient rich foods, food liking, perceived health benefits, and price are often seen as trade-offs (130–133). Several studies indicated that when consumer are more focussed on taste than health they, in general, make unhealthier food choices (18, 131, 133). Steering taste focussed consumers, who are less concerned about health, to healthy food choices is difficult and providing more health related information on food packaging [see (134) for review] is unlikely to solve the problem (135–138). In order to attract the attention of taste focussed consumers it has been recommended to emphasize the great taste of healthy products, rather than to fully focus on the health benefits (16). Alternatively, nutrient poor foods can be made less attractive by increasing its pricing (e.g., sugar tax). Such approach has shown to decrease the purchase of those nutrition poor foods which are taxed in modeling studies, experimental studies as well as natural experiments (139, 140). However, it remains unclear how this strategy will benefit long term healthy food choices and whether consumers are not driven to other unhealthy foods which are not taxed (141).

Future studies should focus on strategies which makes it easier for taste focussed consumers to make long term healthy food choices. In addition it needs to be investigated how the full sensory profiles of foods (e.g., taste, smell, and texture), rather than just taste, are associated with food choices of different segments of the population and how liking for different taste profiles are related to food intake and health outcomes.

In conclusion, the typical taste profile of nutrient poor foods makes them attractive to consumers. The innate liking for sweet and salty taste can make it difficult to move consumers away from nutrient poor foods. However, taste preferences and subsequent food choices can be changed by repeated exposure especially during childhood during which taste preferences play a major role in food choice and consumption. In addition, strategies in which the good taste of nutrient rich foods are emphasized are especially recommended for those consumers who are more taste than health focussed.

## Author Contributions

DL and CR contributed to the outline of this review and writing of the manuscript.

### Conflict of Interest

The authors declare that the research was conducted in the absence of any commercial or financial relationships that could be construed as a potential conflict of interest.
